# Parathyroid hormone-related protein as a potential prostate cancer biomarker: Promoting prostate cancer progression through upregulation of c-Met expression

**DOI:** 10.17305/bb.2023.9753

**Published:** 2024-04-01

**Authors:** Yan Zhao, Zhen-Yu Fu, Han-Yong Feng, Yu-Hao Peng, Zhi-Xiang Yin, Jing-Yi Cao, Chang-Song Pei

**Affiliations:** 1 Department of Urology, The First Affiliated Hospital of Soochow University, Suzhou, China; 2 Department of Urology, Xuzhou Cancer Hospital, Affiliated Hospital of Jiangsu University, Xuzhou, China; 3 Department of Urology, Changshu No. 2 People’s Hospital, Changshu, China

**Keywords:** Parathyroid hormone-related protein (PTHrP), mesenchymal-epithelial transition factor (c-Met), clinicopathological parameter, prostate cancer

## Abstract

Parathyroid hormone-related protein (PTHrP) plays a significant role in various tumor types, including prostate cancer. However, its specific role and underlying mechanisms in prostate cancer remain unclear. This study investigates the role of PTHrP and its interaction with the c-Met in prostate cancer. PTHrP was overexpressed and knocked down in prostate cancer cell lines to determine its effect on cell functions. Xenograft tumor models were employed to assess the impact of PTHrP overexpression on tumor growth. To delve into the interaction between PTHrP and c-Met, rescue experiments were conducted. Clinical data and tissue samples from prostate cancer patients were gathered and analyzed for PTHrP and c-Met expression. PTHrP overexpression in prostate cancer cells upregulates c-Met expression and augments cell functions. In contrast, PTHrP-knockdown diminishes c-Met expression and inhibits cell functions. In vivo experiments further demonstrated that PTHrP overexpression promoted tumor growth in xenograft models. Moreover, modulating c-Met expression in rescue experiments led to concurrent alterations in prostate cancer cell functions. Immunohistochemical analysis of clinical samples displayed a significant positive correlation between PTHrP and c-Met expression. Additionally, PTHrP expression correlated with clinical parameters like prostate-specific antigen (PSA) levels, tumor stage, lymph node involvement, distant metastasis, and Gleason score. PTHrP plays a crucial role in prostate cancer progression by upregulating c-Met expression. These insights point to PTHrP as a promising potential biomarker for prostate cancer.

## Introduction

Prostate cancer is a common male urinary system malignancy, with approximately 1.3 million new cases reported globally each year [1]. Given its high morbidity and mortality, early diagnosis is crucial for better clinical outcomes. While there has been progress in identifying new targets and molecules for managing prostate cancer, there is still a lack of reliable diagnostic and monitoring tools. Thus, the search for dependable biomarkers to enable early diagnosis is an ongoing endeavor.

Parathyroid hormone-related protein (PTHrP) is a peptide extracted and purified from malignant hypercalcemia tumors. It is a polypeptide that is closely associated with cell proliferation, differentiation, and calcium transfer. The PTHrP gene, known as *PTHLH*, is located on the short arm of chromosome 12. Numerous studies have demonstrated a significant correlation between PTHrP and tumor progression, highlighting its ability to promote the occurrence and development of various types of tumors [2, 3]. Likewise, in the context of prostate cancer progression, PTHrP plays a pivotal role in the underlying biology. Current research findings suggest that PTHrP may contribute to the progression of prostate cancer. However, there are also studies suggesting that PTHrP can exert inhibitory effects on prostate cancer by reducing the formation of prostate cancer blood vessels. These divergent results indicate that the precise impact of PTHrP on the progression of prostate cancer remains ambiguous [4–6]. Therefore, the imperative is to delve into the biological efficacy of PTHrP in the context of prostate cancer.

Recent molecular biology research has shed light on the mechanisms by which PTHrP promotes the progression of prostate cancer. These mechanisms include the regulation of epithelial-to-mesenchymal transition (EMT) markers [7], modulation of MAPK and Wnt signaling pathways [8, 9], promotion of prostate cancer angiogenesis [10], and suppression of apoptosis in prostate cancer cells [5]. Despite these significant findings, several aspects of PTHrP’s impact on prostate cancer remain unclear. It is yet to be determined whether PTHrP exerts additional effects on prostate cancer through alternative pathways, and its precise role in clinical prostate cancer progression is still not fully understood. Therefore, further investigation is warranted to elucidate the efficacy of PTHrP in prostate cancer and explore the underlying mechanisms by which it regulates prostate cancer development. Additionally, there is a need to explore the potential of PTHrP as a biomarker for prostate cancer tumors.

The c-Met protein acts as the ligand for hepatocyte growth factor (HGF) and is primarily expressed in epithelial cells. It plays a vital biological role in tumor proliferation, migration, and invasion through its phosphorylation activity [11]. Dysregulation of c-Met has been associated with drug resistance in tumor cells, and the inhibition of c-Met expression is being explored as a potential complementary treatment alongside conventional therapies [12, 13]. In the context of prostate cancer progression, c-Met also holds significant importance. Encouraging results have been observed in numerous preclinical studies targeting c-Met in prostate cancer [14, 15] highlighting its critical role in the disease’s advancement. However, the current understanding of the potential relationship between c-Met and PTHrP in prostate cancer remains unclear. Research findings have suggested potential mechanisms by which c-Met promotes prostate cancer progression, including the Zeb-1 signaling pathway [16], MAPK signaling pathway [17], alterations in the EMT marker expression pathway [18], and activation of the stem cell-related Notch pathway [19]. These findings suggest that PTHrP and c-Met may share common mechanisms in driving prostate cancer progression, indicating a potential cooperative interaction between them. Given the crucial role of c-Met in prostate cancer advancement, establishing the correlation between PTHrP and c-Met would further support the potential of PTHrP as a biomarker for prostate cancer. Therefore, investigating the potential correlation between PTHrP and c-Met in prostate cancer would provide valuable insights and contribute to our understanding of the disease. It may also pave the way for the development of targeted therapies and the utilization of PTHrP as a potential biomarker for prostate cancer.

Recently, an animal experiment study has provided evidence suggesting that the progression of colorectal cancer is regulated by the influence of PTHrP on c-Met [20]. This finding hints at the potential involvement of PTHrP in activating c-Met and contributing to the aggressive behavior of prostate cancer cells. Therefore, the current research aims to elucidate the biomedical functions of PTHrP in prostate cancer cells, explore the potential association between PTHrP and c-Met in prostate cancer, and preliminarily assess the possibility of PTHrP serving as a biomarker for prostate cancer. By shedding light on these aspects, this study seeks to contribute valuable insights to the field of prostate cancer research.

## Materials and methods

### Cell lines and cell culture

The human prostate cancer cells PC3 and DU145 were maintained in our laboratory. PC3 and DU145 cells were separately cultured in sterile conditions using Ham’s F-12 medium (MediaTech, Herndon, VA, USA) supplemented with 10% FBS and 1% penicillin–streptomycin, as well as MEM medium (MediaTech, Herndon, VA, USA). The cells were incubated in a standard culture incubator at 37 ^∘^C with 5% CO_2_ until they reached 80%–90% confluency. Subsequently, they were passaged using 0.25% trypsin digestion. We selected these two cell lines, PC3 and DU145, for our study based on the relatively low basal expression of PTHrP in DU145 cells and the higher basal expression of PTHrP in PC3 cells [21].

### Plasmid construction

The Plvx-copGFP and pcDNA3.1 vectors were purchased from Beijing Qingke Biotechnology Co., Ltd. Total *PTHLH* mRNA and c-Met mRNA were extracted from cells, followed by reverse transcription to generate cDNA using the extracted mRNA as a template. The coding sequences (CDSs) of the target genes were then specifically amplified. The resulting amplification products were analyzed by 1% agarose gel electrophoresis, and the desired bands were purified from the gel. To facilitate cloning, the purified target fragments and vectors were double-digested with restriction enzymes. Subsequently, they were ligated using a rapid ligation enzyme at 25 ^∘^C for 1 h. The ligation products were then transformed into DH5α competent cells and plated on LB agar medium supplemented with ampicillin. After overnight incubation, individual colonies were selected and cultured on a shaker overnight. Plasmids were subsequently extracted. To verify the accuracy of the plasmids, DNA sequencing and site-specific PTHrP immunoassays were performed [22].

### Construction of stable cell lines and cell transfection

The sh-*PTHLH* plasmid was purchased from Beijing Qingke Biotechnology Co., Ltd. *PTHLH* overexpression stable transfection was performed in the DU145 cell line, while *PTHLH*-knockdown stable transfection was performed in the PC3 cell line, along with their corresponding negative control stable transfection strains. The transfection process involved the use of plasmids and subsequent selection with puromycin. Lentiviral particles were generated to package the plasmids, following a method previously described [23]. Following the plating of the cells in 6-well plates, they were allowed to reach 70% confluency before adding the viral supernatant. After a 72-h incubation period, the efficiency of infection was assessed. Subsequently, puromycin selection was initiated to isolate cells that had successfully incorporated the desired genetic material. The selected cells were then expanded in culture. The process involved multiple rounds of selection to ensure the establishment of stable transfected cell lines displaying resistance to the drug.

In addition, selected cell lines were transfected with pcDNA3.1-c-Met, empty pcDNA3.1 plasmid, c-Met small interfering RNA (siRNA) obtained from Yada Fenghui (Hunan, China), and negative control siRNA also obtained from Yada Fenghui (Hunan, China). According to the instructions provided by the manufacturer, Lipofectamine 3000 reagent (Thermo Fisher Scientific, Shanghai, China) was used to perform cell transfection of plasmids and siRNA.

### Quantitative reverse-transcription PCR

Total RNA was isolated with Trizol (Invitrogen, Groningen, The Netherlands). cDNA was synthesized using the Archive Kit (Applied Biosystems, Foster City, CA, USA) according to the manufacturer’s instructions. Real-time PCR reaction mixes were performed using Power SYBR Green (Applied Biosystems, Foster City, CA, USA), and run on the 7500 real-time PCR system (Applied Biosystems) using the following program: 95 ^∘^C for 30 s, 95 ^∘^C for 5 s, 60 ^∘^C for 34 s, 95 ^∘^C for 15 s, 60 ^∘^C for 1 min, and 95 ^∘^C for 15 s, for 40 cycles. The comparative ddCt method was used to analyze results and product specificity was ensured using the melting curves. Experiments were repeated three times independently and done in technical triplicates. GAPDH was used as the internal reference gene. The primers were purchased from Suzhou Jinweizhi Biotechnology Co., Ltd., and their sequences are as follows: *PTHLH* Forward 5’-AAGGTGGAGACGTACAAAGAGC-3’, *PTHLH* Reverse 5’-CAGAGCGAGTTCGCCGTTT-3’, c-Met Forward 5’-AGCGTCAACAGAGGGACCT-3’, c-Met Reverse 5’-GCAGTGAACCTCCGACTGTATG-3’, GAPDH Forward 5’-GGAGCGAGATCCCTCCAAAAT-3’, GAPDH Reverse 5’-GGCTGTTGTCATACTTCTCATGG-3’.

### Western blot analysis

The cells were collected, and total proteins were extracted using protein lysis buffer (KeyGEN BioTECH, Nanjing, China) following the manufacturer’s instructions. Equal amounts of proteins were separated by 10% SDS-PAGE gels using the wet transfer method to transfer the proteins onto PVDF membranes (Millipore, Germany). The membranes were then blocked with 5% skim milk on a shaker at room temperature for 1 h. Subsequently, the membranes were incubated overnight at 4 ^∘^C on a shaker with the respective primary antibodies, including GAPDH (Proteintech, Rosemont, IL, USA), PTHrP (Proteintech, Rosemont, IL, USA), and c-Met (Proteintech, Rosemont, IL, USA) rabbit monoclonal antibodies. After incubation, the membranes were washed three times with TBS-T for 5 min each to remove unbound primary antibodies. Next, the membranes were incubated with anti-rabbit secondary antibodies (Vector Laboratories, Burlingame, CA, USA) for 2 h at room temperature. Protein expression in each group of cells was visualized using a chemiluminescence imaging system.

### Cell scratch assay

DU145 and PC3 cells were digested using trypsin and then suspended. The cell suspension was seeded into a 6-well plate and incubated until the cells covered approximately 90% of the wells. A scratch was created horizontally using a pipette tip, followed by washing with PBS and replacement of the culture medium. The migration of cells at 0 and 24 h after scratching was observed and recorded using a microscope from Olympus, Japan. To determine the migration rate, the length between the two boundaries of eight randomly selected scratches was measured using Image J software.

### Cell proliferation assay

The cell suspension was diluted to a concentration of 2×10^4^ cells/mL. Next, 100 µL of the diluted cell suspension was added to each well of a 96-well plate. The plate was then placed inside a cell culture incubator. After 24, 48, 72, and 96 h, the respective plates were removed from the incubator. To assess cell viability, 10 µL of Cell Counting Kit-8 (CCK-8; Dojindo, Kyushu, Japan) was added to each well, followed by a 2-h incubation period. The optical density (OD) values of each well were measured at a wavelength of 450 nm using an enzyme-labeled instrument.

### 5-Ethynyl-2′-deoxyuridine (EdU) proliferation assay

To display the function of PTHrP or c-Met on cell proliferative, the EdU proliferation assay (Beyotime, Shanghai, China) was conducted following the guidelines provided by the manufacturer. The cells were exposed to a 50 µM EdU solution for a period of 2 h. Following this, a click reaction was conducted, and the cells were incubated with Hoechst 33,342. Finally, the cell images were acquired using a fluorescence microscope. The proportion of EdU-positive cells, which corresponds to proliferating cells, was determined by calculating the ratio of Azide 555-labeled cells (appearing as red) to the sum of Azide 555-labeled cells (red) and Hoechst-labeled cells (blue).

### Matrigel invasion assay

The invasion ability of DU145 and PC3 cells was assessed using the Transwell cell invasion assay with 8 mm pores (Corning, NY, USA) in 24-well plates. In the upper chamber, 10 µL of Matrigel gel (Becton Dickinson, Bedford, MA, USA) diluted 1:7 in serum-free culture medium was added. The plates were then incubated at 37 ^∘^C in a cell culture incubator for 1 h to allow the Matrigel gel to solidify. Cell counting was performed, and a suspension of 5×10^4^ cells in 200 µL of serum-free culture medium was added to the upper chamber of the Transwell insert. The lower chamber was filled with 500 µL of culture medium containing 10% FBS. After 24 h, the liquid in the upper chamber was removed, and the cells on the upper surface were gently wiped off using a cotton swab. The cells were fixed with paraformaldehyde, stained with crystal violet, washed with PBS, air-dried, and then observed and counted under an inverted microscope. This experimental procedure was repeated three times.

### In vivo experiments

The Animal Care and Use Committee of Soochow University authorized all experimental animal procedures, ensuring the use of specific pathogen-free (SPF) conditions for animal rearing. BALB/c male nude mice, five weeks of age or older, were obtained from the Animal Center of Soochow University in Suzhou, China. In each group, a total of 2 × 10^6^ DU145 cells suspended in 100-µL PBS, either stably transfected with *PTHLH* or vector, were subcutaneously injected into the mice axillae (*n* ═ 6). After a period of four weeks following inoculation, all mice were killed, and the subcutaneous tumor tissues were carefully harvested and weighed. Tumor volume was determined using the formula: length × width^2^ × 0.5. Next, the tumor tissues were preserved by fixation in 10% neutral phosphate-buffered formalin. Following fixation, dehydration, clearing, and wax immersion, the samples were embedded in paraffin for further analysis and examination.

### Patients and tissue samples

A total of 88 patients diagnosed with prostate cancer were included in this study, conducted at the First Affiliated Hospital of Soochow University between January 2017 and December 2019. The diagnosis of prostate cancer was confirmed through histopathological examination of prostate biopsy or surgical specimens, guided by transrectal ultrasound or radical prostatectomy. Patients who had received prior androgen deprivation therapy, chemotherapy, or radiation therapy were excluded, as well as those with concurrent malignancies. Various diagnostic procedures, including histopathological examination, transrectal ultrasound, prostate-specific antigen (PSA) testing, digital rectal examination, MRI, CT, or bone scans, were utilized for disease grading and staging. Gleason grading followed the system based on the International Society of Urological Pathology consensus conference [24]. Relevant clinical data of the patients were collected and subjected to statistical analysis. Additionally, paraffin sections of postoperative or biopsy primitive carcinoma tissue were collected for immunohistochemical testing.

### The Cancer Genome Atlas data acquisition

The genomic alteration data for patients with prostate cancer and their clinicopathologic profiles were obtained from the publicly accessible The Cancer Genome Atlas (TCGA) (https://cancergenome.nih.gov/). TCGA serves as a valuable platform that provides a wealth of cancer-related data. This study adhered to the publication guidelines and policies set forth by TCGA (https://www.cancer.gov/ccg/research/genome-sequencing/tcga/publications). Therefore, the expression levels of *PTHLH* and c-Met in prostate cancer were analyzed using the TCGA database. We divided *PTHLH* into high- and low-expression groups using the median as the threshold.

### Hematoxylin and eosin staining

Four weeks after tumor implantation, nude mice were euthanized, and subcutaneous tumor tissues were collected. Concurrently, biopsy or postoperative samples of prostate cancer were obtained from clinical patients. The collected tissues were embedded in paraffin and sectioned into 4-µm slices. After deparaffinization using xylene, the sections underwent a series of ethanol washes (absolute ethanol, 95%, 85%, and 70% ethanol) for hydration. Subsequently, the sections were stained with hematoxylin for 10 min followed by eosin for 3 min. The resulting histological changes in prostate cancer tissues were observed under an Olympus CX-43 light microscope.

### Immunohistochemistry analysis

Four weeks after tumor implantation, the expression levels of Ki67 and CD31 were assessed in subcutaneous prostate cancer tissues from nude mice. Additionally, biopsy or postoperative samples obtained from clinical prostate cancer patients were evaluated for the expression levels of PTHrP and c-Met. The collected tissues were fixed in 10% formalin and underwent routine processing before being embedded in paraffin. The paraffin blocks were then sliced into 4 µm sections. Immunohistochemical staining was performed on these sections following a previously described protocol [25]. Briefly, sections were incubated overnight at 4 ^∘^C with primary antibodies, including Ki-67 antibody (dilution 1:8000, Proteintech, Rosemont, IL, USA), CD31 antibody (dilution 1:5000, Proteintech, Rosemont, IL, USA), PTHrP antibody (dilution 1:200, Proteintech, Rosemont, IL, USA), or c-Met antibody (dilution 1:70, Proteintech, Rosemont, IL, USA). After washing the sections three times with phosphate-buffered saline (Gibco-BRL, Gaithersburg, MD, USA), they were incubated with goat anti-rabbit or mouse IgG (Vector Laboratories, Burlingame, CA, USA) for 30 min. Subsequently, the sections were stained using the color reagent 3,3′-diaminobenzidine. Two senior pathologists performed a double-blind review of the entire slide. If there was a discrepancy in their diagnostic opinions, a third pathologist provided a final diagnosis based on the consensus of two concurring opinions. Scoring method: The intensity of cellular staining was scored as follows: 0 (no staining at all), 1 (weak), 2 (medium), or 3 (strong). The percentage of positively stained cells was scored as follows: 0 (0%–5%), 1 (5%–25%), 2 (26%–75%), or 3 (75%–100%). The two scores were summed to obtain the total score. A total score greater than 3 indicated strong expression, while a score of ≤ 3 indicated weak expression [26]. Here, we defined strong expression as high expression and weak expression as low expression.

### Ethical statement

This study was reviewed and approved by the Ethics Committee of the First Affiliated Hospital of Soochow University (FASU20220426x). All study potential risks and procedures have been explained to the patients, and they gave their written informed consent.

### Statistical analysis

The data were expressed as mean ± standard deviation (SD). Continuous variables were analyzed using the two-sample independent *t*-test (two-tailed) or one-way analysis of variance (ANOVA) as appropriate. For categorical variables, the chi-squared (χ^2^) test or Fisher’s exact test was applied. Pearson correlation analysis was conducted to examine the associations between variables. A *P* value less than 0.05 was considered statistically significant. Statistical analysis was performed using SPSS 21.0 software.

**Figure 1.**
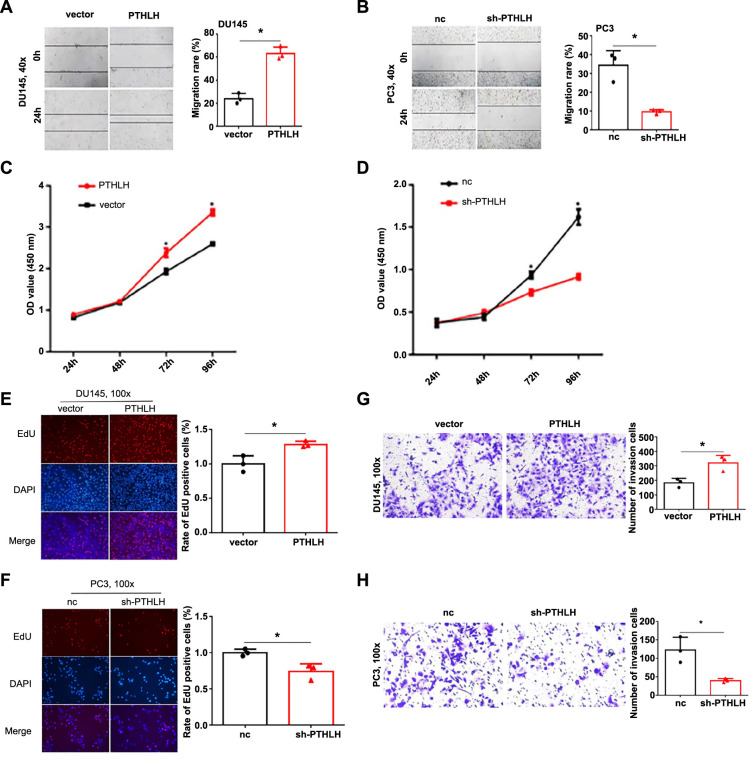
**Parathyroid hormone-related protein boosts prostate cancer cell activity in vitro.** *P < 0.05; (A and B) Scratch assay showed that PTHrP enhances migration of prostate cancer cells; (C–F) CCK-8 and EdU proliferation assays compared proliferation of *PTHLH*-overexpressing DU145 stable cells and *PTHLH*-knockdown PC3 stable cells with control; (G and H) Transwell invasion assay assessed cell invasion in *PTHLH*-overexpressing and *PTHLH*-silenced prostate cancer cells. PTHrP: Parathyroid hormone-related protein; EdU: 5-Ethynyl-2′-deoxyuridine; nc: Negative control; PTHLH: Parathyroid hormone-like hormone gene.

## Results

### PTHrP enhances prostate cancer cell proliferation and invasion in vitro

The biological effects of PTHrP on prostate cancer are currently not well understood. To gain further insight into the role of PTHrP in prostate cancer cells, we conducted subsequent research using DU145 cells (with lower PTHrP expression) and PC3 cells (with higher PTHrP expression). To achieve this, we successfully generated stable cell lines overexpressing *PTHLH* in DU145 cells and knocked down *PTHLH* expression in PC3 cells. Through scratch assays, we observed that overexpression of *PTHLH* increased the migratory ability of prostate cancer cells, whereas *PTHLH*-knockdown resulted in reduced cell migration (Figure 1A and 1B). In order to investigate cell proliferation, we conducted CCK-8 and EdU proliferation assays. The results demonstrated that DU145 cells overexpressing *PTHLH* exhibited enhanced proliferation, while the *PTHLH* inhibition group showed a marked decrease in cell proliferation (Figure 1C–1F), indicating that *PTHLH* promotes the proliferation of prostate cancer cells. Furthermore, Transwell cell invasion assays revealed that *PTHLH* overexpression significantly increased the invasion ability of DU145 cells, whereas the downregulation of *PTHLH* had the opposite effect (Figure 1G and 1H). These findings collectively indicate that *PTHLH* plays a role in promoting the progression of prostate cancer in vitro.

**Figure 2.**
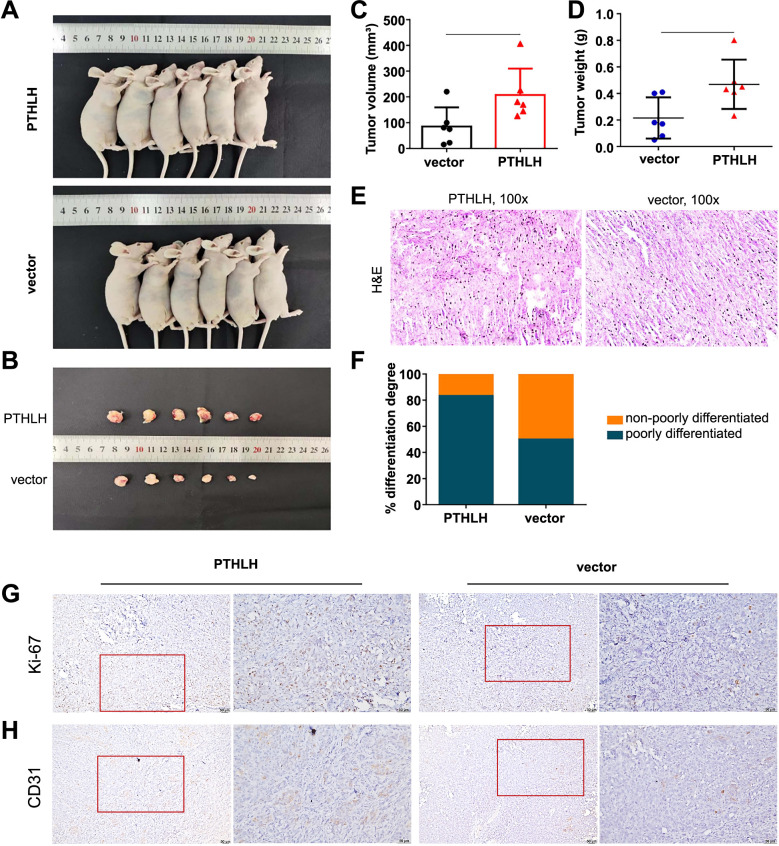
**Parathyroid hormone-related protein drives prostate cancer progression in vivo.** *P < 0.05; (A and B) Illustrations depict subcutaneous xenograft tumors from DU145 cells transfected with *PTHLH* and vector; (C and D) Tumor volumes and weights in DU145 cells were assessed; (E and F) H&E staining of xenograft tumors showed increased poorly-differentiated cancer in the *PTHLH*-overexpressing group; (G and H) IHC analysis revealed Ki-67 and CD31 expression in xenograft tumors. IHC: Immunohistochemistry; H&E: Hematoxylin and eosin; PTHLH: Parathyroid hormone-like hormone gene.

### PTHrP promotes prostate cancer growth in vivo

To investigate the in vivo functions of PTHrP, we conducted a study where we subcutaneously injected DU145 cells transfected with vector and *PTHLH* into nude mice. After a period of four weeks, the mice were euthanized, and the subcutaneous tumor tissues were carefully isolated and subjected to measurements of weight and volume. Notably, our results demonstrated a significant increase in tumor growth when PTHrP was overexpressed (Figure 2A–2D). To gain further insights into the histopathological characteristics of the isolated tumor tissues, we performed hematoxylin and eosin (H&E) staining, which revealed a higher proportion of poorly differentiated tumors in the PTHrP overexpression group compared to the vector group (Figure 2E and 2F). Additionally, the immunohistochemistry (IHC) analysis showed an upregulation of the Ki-67 antigen, indicative of enhanced cellular proliferation, in the PTHrP-overexpressing xenograft tumors (Figure 2G). Moreover, IHC staining of the tumor tissues revealed increased CD31 expression in the PTHrP overexpression group, suggesting that PTHrP may promote tumor angiogenesis in vivo (Figure 2H). Collectively, our findings provide compelling evidence that PTHrP overexpression actively drives the in vivo setting growth of prostate cancer.

### Enhanced c-Met expression induced by PTHrP in prostate cancer cells

**Figure 3.**
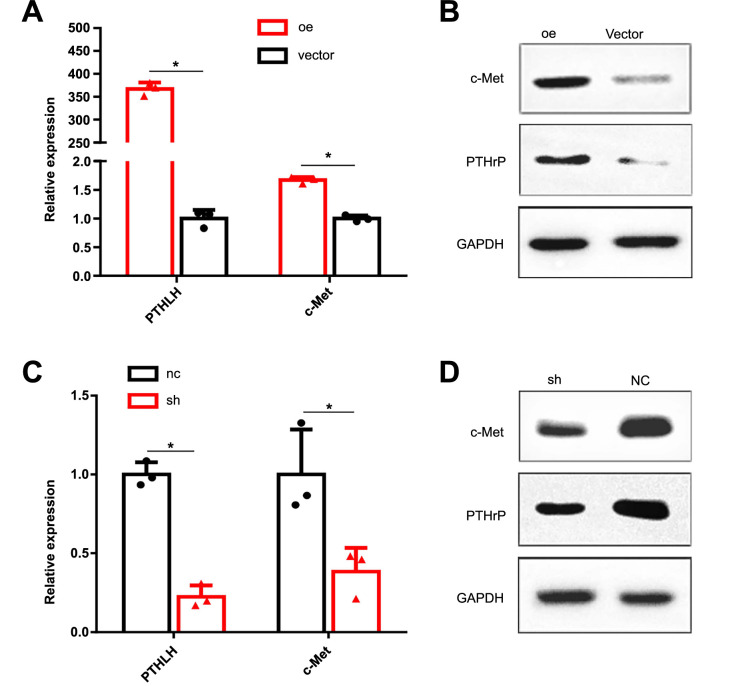
**Parathyroid hormone-related protein increases c-Met expression in prostate cancer cells.** *P < 0.05; (A and B) PTHrP and c-Met mRNA and protein levels were assessed in *PTHLH*-overexpressing DU145 stable transfectants using qRT-PCR and western blot analysis; (C and D) PTHrP and c-Met mRNA and protein levels were analyzed in *PTHLH*-knockdown PC3 stable transfectants using qRT-PCR and western blot analysis. PTHrP: Parathyroid hormone-related protein; PTHLH: Parathyroid hormone-like hormone gene.

To investigate the relationship between PTHrP and c-Met in prostate cancer cells, we chose DU145 cells (with lower PTHrP expression) and PC3 cells (with higher PTHrP expression) for further analysis. Interestingly, we observed a significant increase in both mRNA and protein levels of c-Met upon *PTHLH* overexpression, while knockdown of *PTHLH* resulted in a decrease in c-Met levels (Figure 3). These findings collectively provide compelling evidence for the correlation between the oncogene c-Met and PTHrP, with PTHrP playing a role in promoting the expression of c-Met.

**Figure 4.**
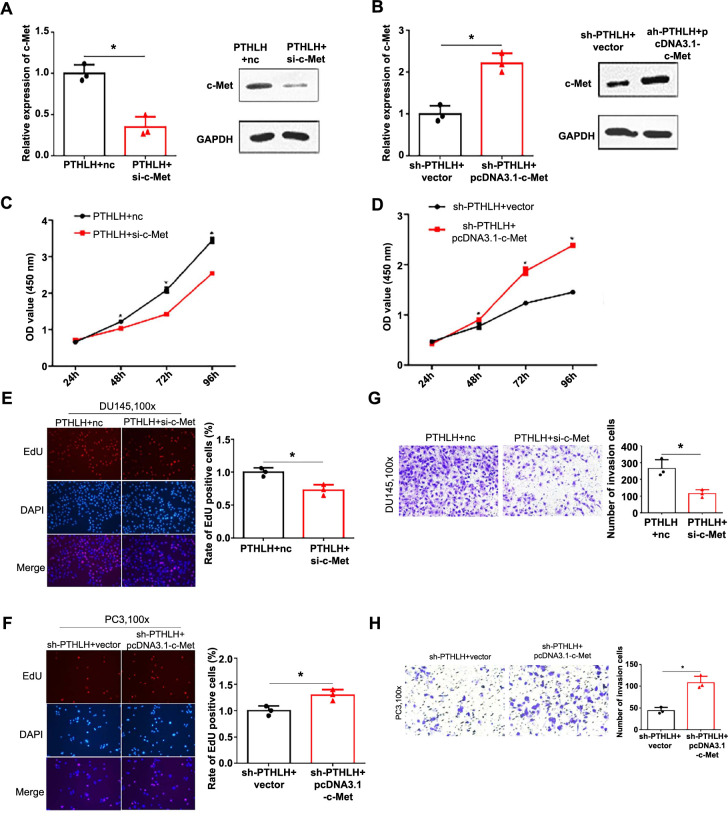
**Parathyroid hormone-related protein enhances prostate cancer cell functions via c-Met modulation.** *P < 0.05; (A and B) c-Met expression was verified by qRT-PCR and western blot analysis in transfected cell lines; (C–F) The impact of PTHrP and c-Met on prostate cancer cell proliferation was assessed using CCK-8 and EdU assays in DU145 and PC3 cells; (G and H) Transwell invasion assay evaluated the influence of PTHrP and c-Met on invasive potential in DU145 and PC3 cells. CCK-8: Cell Counting Kit-8; EdU: 5-Ethynyl-2′-deoxyuridine; PTHLH: Parathyroid hormone-like hormone gene.

**Figure 5.**
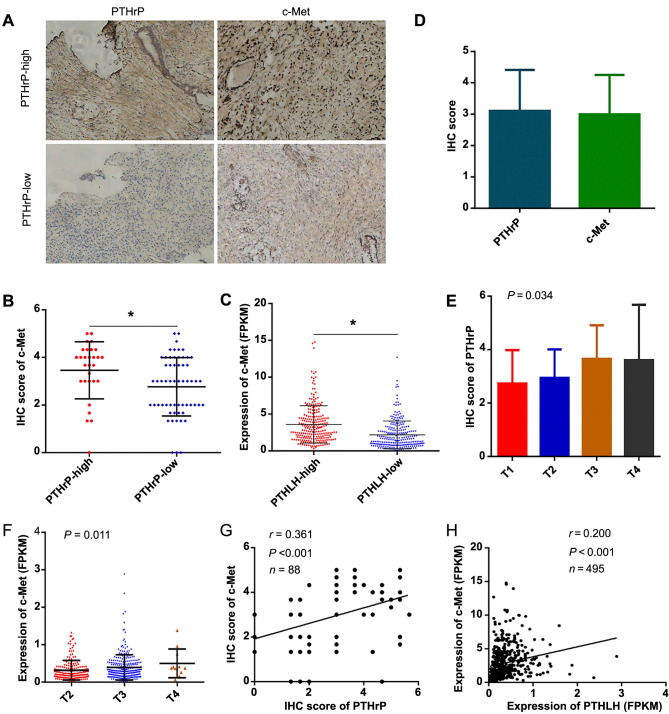
**Parathyroid hormone-related protein in clinical prostate cancer tissue analysis.** *P < 0.05; (A and B) High PTHrP expression in prostate cancer tissues correlated with increased c-Met expression; (C) c-Met expression significantly upregulated in clinical prostate cancer tissues with high *PTHLH* expression compared to low expression in TCGA data; (D) IHC scores for PTHrP and c-Met in prostate cancer tissues; (E–H) PTHrP IHC scores increased with advanced T-stage, and a significant positive correlation was observed between PTHrP and c-Met IHC scores in clinical prostate cancer tissue and TCGA database, respectively. IHC: Immunohistochemistry; PTHrP: Parathyroid hormone-related protein; TCGA: The Cancer Genome Atlas; c-Met: Mesenchymal-epithelial transition factor; PTHLH: Parathyroid hormone-like hormone gene.

### PTHrP enhances the biological functions of prostate cancer cells by modulating c-Met

To investigate whether the promotion of biological functions induced by aberrant expression of PTHrP is associated with the expression of c-Met in prostate cancer cells, we performed rescue assays. In order to downregulate c-Met expression, we employed siRNA in DU145 cells, while PC3 cells were transfected with pcDNA3.1-c-Met to upregulate c-Met expression. The expression of c-Met was evaluated at both the mRNA and protein levels using qRT-PCR and western blot analysis, respectively. Our results demonstrated that PTHrP overexpression in DU145 cells transfected with si-c-Met led to a decrease in c-Met expression compared to the control group. Conversely, in PC3 cells with downregulated PTHrP and transfected with pcDNA3.1-c-Met, there was an observed increase in the relative expression of c-Met (Figure 4A and 4B). Furthermore, the downregulation of c-Met in DU145 cells significantly inhibited cell proliferation and invasion capabilities (Figure 4C, 4E, and 4G), while the overexpression of c-Met in PC3 cells significantly enhanced cell proliferation and invasion abilities (Figure 4D, 4F, and 4H). Overall, these findings suggest that PTHrP promotes the proliferation and invasion capabilities of prostate cancer cells by modulating c-Met.

### Characterization of PTHrP in clinical prostate cancer tissue

To investigate the role of PTHrP, the expression levels of PTHrP and c-Met were assessed in clinical prostate cancer tissue. Our findings revealed that among 88 cases of prostate cancer tissue, with clinical information provided in Table 1, the IHC score for PTHrP was 3.11 ± 1.31, while the IHC score for c-Met was 2.99 ± 1.26. Prostate cancer tissues with high PTHrP expression exhibited elevated c-Met expression (*P* ═ 0.014). Additionally, we analyzed the TCGA database and found that the expression level of c-Met was significantly upregulated in clinical tissues with high *PTHLH* expression compared to those with low *PTHLH* expression (*P* < 0.001), as shown in Figure 5A–5D. Furthermore, in clinical prostate cancer tissue, the IHC score of PTHrP increased with advanced T-stage (*P* ═ 0.034), and there was a significant positive correlation between the IHC scores of PTHrP and c-Met (*r* ═ 0.361, *P* < 0.001), as depicted in Figure 5E and 5G. These findings were consistent with the data obtained from the TCGA database (Figure 5F and 5H).

**Table 1:** Comprehensive clinical profile of the 88 prostate cancer patients

**Characteristics**	**PTHrP expression**		***P* value**
	**High**	**Low**	
Age, years (range)	55 – 95	52 – 87	0.545
Type of samples (*n*, %)			0.001
Surgical specimen	6 (6.8%)	36 (41.0%)	
Biopsies	23 (26.1%)	23 (26.1%)	
Gleason score (*n*, %)			0.111
≤ 6	3 (3.4%)	19 (21.6%)	
7 (3+4)	2 (2.3%)	8 (9.1%)	
7 (4+3)	5 (5.7%)	6 (6.8%)	
8	9 (10.2%)	15 (17.0%)	
9 and 10	10 (11.4%)	11 (12.5%)	
pT stage (*n*, %)			0.001
T1	5 (5.7%)	30 (34.1%)	
T2	6 (6.8%)	16 (18.2%)	
T3	14 (15.9%)	12 (13.6%)	
T4	4 (4.5%)	1 (1.2%)	
cN stage, *n* (%)			0.010
N0	13 (14.8%)	43 (48.8%)	
N1	16 (18.2%)	16 (18.2%)	
M stage (*n*, %)			0.001
M0	2 (2.3%)	21 (23.9%)	
M1a	2 (2.3%)	6 (6.8%)	
M1b	12 (13.6%)	28 (31.8%)	
M1c	13 (14.8%)	4 (4.5%)	
Initial PSA, ng/mL (range)	2.9 – 5654	2.5 – 2689	0.115
ECOG score, mean	1.034 ± 1.052	0.881 ± 0.892	0.478

PTHrP: Parathyroid hormone-related protein; PSA: Prostate-specific antigen; ECOG: Eastern Cooperative Oncology Group.

Statistical analyses, including the χ^2^-test and Fisher’s exact test, were employed to investigate the potential relationship between PTHrP expression levels and clinicopathological characteristics in a cohort of 88 prostate cancer patients. The results demonstrated a positive correlation between PTHrP expression and several clinicopathological features, including initial PSA level (*P* ═ 0.026), pT stage (*P* ═ 0.001), lymph node metastasis (*P ═* 0.010), distant metastasis (*P* ═ 0.004), and Gleason score (*P* ═ 0.026). Notably, no significant correlation was observed between PTHrP expression and patient age (*P* ═ 0.729), as detailed in Table 2. These findings imply that PTHrP may exert a promoting influence in the context of clinical prostate cancer.

**Table 2:** The correlations between parathyroid hormone-related protein in tissues and clinicopathologic features of prostate cancer patients

**Characteristics**		*n*	**High-expression PTHrP *n (%)***	**Low-expression PTHrP *n (%)***	*P* **value**
Age (years)	≤ 65	17	5 (29.4%)	12 (70.6%)	0.729
	> 65	71	24 (33.8%)	47 (66.2%)	
Initial PSA (ng/mL)	≤ 10	19	2 (10.5%)	17 (89.5%)	0.026
	> 10	69	27 (39.1%)	42 (60.9%)	
pT stage	T1	35	5 (14.3%)	30 (85.7%)	0.001
	T2	22	6 (27.3%)	16 (72.7%)	
	T3	26	14 (53.8%)	12 (46.2%)	
	T4	5	4 (80.0%)	1 (20.0%)	
cN stage	N0	56	13 (23.2%)	43 (76.8%)	0.010
	N1	32	16 (50.0%)	16 (50.0%)	
M stage	M0 and M1a	31	4 (12.9%)	27 (87.1%)	0.004
	M1b and M1c	57	25 (43.9%)	32 (56.1%)	
Gleason score	≤ 6	22	3 (13.6%)	19 (86.4%)	0.026
	≥ 8	45	19 (42.2%)	26 (57.8%)	
Total		88	29 (32.9%)	59 (67.1%)	

PTHrP: Parathyroid hormone-related protein; PSA: Prostate-specific antigen.

## Discussion

Prostate cancer is one of the most commonly encountered malignant tumors in the male urinary system. Globally, there are approximately 1.3 million new cases of prostate cancer reported each year [1]. Prostate cancer exhibits a persistently high mortality rate, as its tendency to metastasize significantly lowers the survival rates [27]. Although clinically utilized parameters, such as serum PSA, Gleason score, and clinical staging, play a crucial role in assessing the prognosis of prostate cancer, their predictive efficacy remains suboptimal. Studies have indicated that exosomal biomarkers, TKTL1, and UHRF1 are associated with the prognosis of prostate cancer and hold potential as tumor markers [28–30]. This suggests the potential existence of more valuable and novel tumor markers for prostate cancer.

PTHrP was initially isolated and identified in patients with malignant hypercalcemia. It is expressed in various tissues, such as skin, bone marrow, and brain, primarily through autocrine or paracrine mechanisms [31, 32]. PTHrP plays a role in the development of several tumors and is associated with tumor metastasis and poorer prognosis [33]. Studies have reported that PTHrP regulates the growth of prostate cancer cells through autocrine/paracrine and intracellular signaling pathways [34]. Additionally, the adhesion capacity of tumor cells to extracellular matrix proteins is crucial for tumor invasion and metastasis. Shen et al. [35] discovered that overexpressing PTHrP in prostate cancer cells enhances their adhesion to extracellular matrix proteins, such as collagen type I, laminin, and fibronectin, suggesting a promoting effect of PTHrP on prostate cancer cell invasion and metastasis. However, other reports suggest that PTHrP can reduce the expression of vascular endothelial growth factor (VEGF) and inhibit tumor angiogenesis in prostate cancer through the activation of protein kinase A (PKA), thereby suppressing tumor growth [4, 36]. These conflicting research results may be due to the diversity within the tumor microenvironment and also indicate that the precise biological functions of PTHrP in prostate cancer cells remain unclear. Therefore, this study aimed to investigate the impact of PTHrP overexpression and knockdown on the biological functions of prostate cancer cells and their influence on tumor growth. Our findings reveal that PTHrP enhances the migration, invasion, and proliferation capabilities of prostate cancer cells, thereby promoting tumor growth. Consequently, it is intriguing to further explore whether PTHrP could serve as a potential biomarker for prostate cancer.

Previous studies have attempted to explore the impact of PTHrP on clinical prostate cancer. Bryden et al. [37] conducted a study and found that both primary and metastatic prostate cancer lesions express PTHrP, with higher expression levels observed in the metastatic lesions. Additionally, reports indicate that plasma PTHrP levels in prostate cancer patients are correlated with the aggressiveness of the tumor. Higher malignancy grades are associated with elevated PTHrP expression levels. The detection sensitivity of PTHrP ranges from 83% to 91%, with a specificity ranging from 78% to 96% [38]. These findings suggest a strong association between PTHrP and clinical prostate cancer. However, there is currently a lack of research on the specific expression patterns of PTHrP in prostate cancer tissue and its relationship with clinical pathological parameters. Therefore, this study aimed to investigate these aspects. In previous studies, a Gleason score of ≤6 was considered indicative of indolent cancer, while a Gleason score of ≥8 indicated more aggressive forms [39, 40]. In the current study, we observed a distinct difference in PTHrP expression between Gleason scores ≤6 and ≥8 in primary prostate cancer tissues. This suggests that PTHrP may have the potential to differentiate the malignancy grade of clinical prostate cancer. Moreover, bone metastasis is a significant milestone in the progression of prostate cancer and represents a systemic disease that greatly affects patient prognosis. Once bone metastasis occurs, prostate cancer patients may experience symptoms, such as bone pain, pathological fractures, and spinal cord compression, ultimately leading to a reduced quality of life and shortened lifespan [41]. Our findings indicated that PTHrP expression in tissues was significantly higher in phases M1b and M1c compared to phases M0 and M1a, implying that PTHrP may play a role in the bone metastasis process in clinical prostate cancer patients. Furthermore, we assessed the relationship between PTHrP and other clinical pathological parameters. Our results revealed a close correlation between PTHrP expression in primary tumor tissues and positive lymph node metastasis, pathological *T* stage, and high initial PSA levels. Another recent study showed that pancreatic cancer patients with high PTHrP expression in tumor tissues had a poorer survival prognosis, suggesting that PTHrP within tumor tissues might serve as a tumor marker for pancreatic cancer [42]. Taking our findings into consideration, we propose that PTHrP could potentially serve as a novel tumor marker for prostate cancer. However, further clinical research is necessary to validate it.

According to the available information, it is suggested that PTHrP may play a role in the progression of prostate cancer. However, the specific mechanisms involved in this process have not been fully elucidated. Several studies have indicated that c-Met, a known factor in prostate cancer progression, shares some mechanisms with PTHrP in promoting tumor advancement [7, 8, 14, 15, 17, 18]. This suggests a potential synergistic effect between PTHrP and c-Met during prostate cancer development. Supporting this notion, research conducted by Novoa Díaz et al. [20] demonstrated that PTHrP can influence tumor biology by regulating c-Met in colorectal cancer cells. Based on these findings, it can be inferred that PTHrP-mediated modulation of c-Met expression may contribute to the promotion of prostate cancer progression. To investigate this hypothesis, this study employed overexpression and interference techniques targeting the *PTHLH* gene in different prostate cancer cells. The results revealed that PTHrP overexpression led to an increase in c-Met expression and enhanced biological functions of the tumor cells. Conversely, interference with PTHrP resulted in decreased c-Met expression and weakened tumor cell activity. The observed reversal of tumor cell biological functions upon manipulation of c-Met expression further supports the notion that PTHrP’s pro-prostate cancer effects are at least partially mediated by its influence on c-Met activity and expression levels. Furthermore, in clinical prostate cancer patients, it was observed that those with high expression of PTHrP displayed significantly elevated levels of c-Met expression compared to individuals with low PTHrP expression. Moreover, there was a positive correlation between the expression levels of PTHrP and c-Met, providing additional evidence for the potential existence of the PTHrP/c-Met pathway axis in prostate cancer.

This study had some limitations, including the fact that prostate cancer is considered a slow-growing tumor with a relatively long survival period after diagnosis. As a result, we did not collect data on survival indicators, such as overall survival and progression-free survival for prostate cancer patients, nor did we conduct an analysis of the impact of PTHrP expression on the survival of prostate cancer patients. Furthermore, in our animal experiments, we only established a subcutaneous tumor model in nude mice, which demonstrated the influence of PTHrP on local prostate cancer growth. We did not further investigate the role of PTHrP in prostate cancer metastasis, and future research is needed to confirm this aspect. Finally, this study included a relatively small sample of 88 clinical cases, and larger-scale studies are warranted in the future.

Studies have provided evidence that the cholesterol-lowering medication, simvastatin, not only has inhibitory effects on prostate cancer [43] but also specifically targets PTHrP [44]. These findings suggest a potential association between PTHrP-mediated prostate cancer progression and lipid metabolism, highlighting the significant role of PTHrP in the advancement of prostate cancer. Utilizing PTHrP as a therapeutic target for prostate cancer treatment holds promise, although further experimental verification is necessary to validate its efficacy.

## Conclusion

Our study elucidates the crucial role of PTHrP in driving the progression of prostate cancer. Specifically, we indicated that PTHrP-mediated dysregulation of c-Met may contribute significantly to the advancement of prostate cancer. These novel findings provide valuable insights into the oncogenic properties of PTHrP in prostate cancer and underscore its potential as a promising therapeutic target, potentially offering new approaches to the diagnosis and treatment of prostate cancer.

## Data Availability

The data are contained within this article.
